# Exosomics

**DOI:** 10.14806/ej.26.0.934

**Published:** 2020-08-04

**Authors:** Thanasis Mitsis, Katerina Pierouli, Kalliopi lo Diakou, Eleni Papakonstantinou, Flora Bacopoulou, George P. Chrousos, Dimitrios Vlachakis

**Affiliations:** 1 Laboratory of Genetics, Department of Biotechnology, School of Applied Biology & Biotechnology, Agricultural University of Athens, Athens, Greece; 2 University Research Institute of Maternal and Child Health & Precision Medicine, and UNESCO Chair on Adolescent Health Care, National and Kapodistrian University of Athens, “Aghia Sophia” Children’s Hospital, Athens, Greece; 3 Division of Endocrinology and Metabolism, Center of Clinical, Experimental Surgery and Translational Research, Biomedical Research Foundation of the Academy of Athens, Athens, Greece

## Abstract

Extracellular vesicles have been the focus of a large number of studies in the past five years. Exosomes, a subgroup of extracellular vesicles, are of particularly high interest because they partake in a wide number of biological pathways. Produced by a variety of cells, exosomes have an important role in both physiological and pathological conditions. Exosome cargo heavily defines the vesicles’ unique characteristics, and the cargo with the most intriguing prospects in its’ biomedical applications is the non-coding RNAs. Non-coding RNAs, and specifically microRNAs are implicated in the regulation of many biological processes and have been associated with numerous diseases. Exosomes containing such important cargo can be used as biomarkers, therapeutic biomaterials, or even drug carriers. The potential media use of exosomes seems promising. However, some obstacles should be overcome before their clinical application. Synthetic exosome-like biomolecules may be a solution, but their production is still in their beginning stages. This review provides concise information regarding the current trends in exosome studies.

## Introduction

Extracellular Vesicles (EVs) are membrane-bound vesicles secreted by cells into the extracellular space and have the ability to transport various molecules, such as DNA, RNA, and proteins, between cells (Zaborowski et al., 2015; Doyle and Wang, 2019). EVs are, thus, essential mediators of cell-cell communication (Goran Ronquist, 2019; Raposo and Stahl, 2019). They can be classified into three main classes, which are microvesicles, exosomes, and apoptotic bodies (Yáñez-Mó et al., 2015).

Currently, one of the most investigated classes of EVs is exosomes. Exosomes are single-membrane nano-sized vesicles with a diameter of ~30 to ~200nm with a topology similar to that of a cell (Pegtel and Gould, 2019). The reason for the intensive research that has taken place on exosomes is their specific role in cell communication. Intercellular communication through exosomes is important in both physiological and pathological biological function in humans (Camussi et al., 2010). Exosomes appear to be of high importance in development, immunity, homeostasis, cancer, viral replication, tissue regeneration, and neurodegenerative diseases (de la Torre Gomez et al., 2018; Pegtel and Gould, 2019). These abilities that exosomes possess showcase potential clinical applications, both as biomarkers and as therapeutic substance carriers (Zhang et al., 2019).

As mentioned above, EVs can carry different types of molecules between cells. One exosome cargo that has attracted much attention for its potential clinical applications is the non-coding RNA (ncRNA), predominantly microRNAs (miRNAs) (Gallo et al., 2012; Silva and Melo, 2015; Beuzelin and Kaeffer, 2018; Wang et al., 2019). MiRNAs are a class of endogenous ncRNA molecules, approximately 20-22nt in length (Huang et al., 2011) that have significant applicability as possible drug targets, modulators of drug resistance, and biomarkers for a wide variety of pathological conditions (Hanna et al., 2019). The above information implies that modifications in the miRNA cargo of exosomes can provide various benefits in human health and an alternative to traditional drugs (Li et al., 2018). Furthermore, synthetic exosome-mimics can be produced on a large scale, thus providing a feasible platform for a novel method of drug delivery (Li et al., 2018).

## Exosome Function

Exosomes are produced and released by various types of cells in the body, such as nervous system cells, such as Schwann cells (Ge et al., 2012), astrocytes and neurons (Faure et al., 2006; Mignot et al., 2006), by epithelial cells, by fibroblasts and adipocytes, as well as by cells of the immune and hematopoietic systems, where their secretion was first detected,in particular by reticulocytes (Johnstone et al., 1989; Fader et al., 2005; Mignot et al., 2006), B lymphocytes, T cells (Raposo et al., 1996; Laulagnier et al., 2004; Clayton et al., 2005; Chaput et al., 2006; Mignot et al., 2006), platelets (Zitvogel et al., 1998; Heijnen et al., 1999; Laulagnier et al., 2004; Clayton et al., 2005; Chaput et al., 2006; Mignot et al., 2006), mast cells (Raposo et al., 1996; Zitvogel et al., 1998; Skokos et al., 2003; Laulagnier et al., 2004; Clayton et al., 2005; Chaput et al., 2006; Mignot et al., 2006), dendritic cells (Raposo et al., 1996; Zitvogel et al., 1998; Chaput et al., 2006; Mignot et al., 2006), and macrophages (Nguyen et al., 2003; Skokos et al., 2003). Exosomes have also been detected in many types of biological fluids, such as breast milk, amniotic fluid, urine, blood, semen, bronchoalveolar lavage, synovial fluid and in the cerebrospinal fluid (Qin and Xu, 2014; Ellwanger et al., 2017; Isola and Chen, 2017). Nowadays, it has been proven that the primary function of exosomes is the communication between cells, especially when they are distant from each other. Specifically, exosomes move from one cell that secretes them to another cell that internalises them, thereby transferring proteins and genetic material. Exosomes are also capable of transferring and spreading pathogens between cells, such as viruses and prions (Qin and Xu, 2014).

Due to the presence of exosomes in most cell types, they are involved in various procedures both in physiological and pathological conditions. One of the most important processes in which exosomes participate is the immune response. Immune cells secrete exosomes that are responsible for their inter-communication (Raposo et al., 1996). From the beginning of an organism’s infection, cells that recognise antigens, such as dendritic cells (DCs), are responsible for presenting the antigen to other immune cells. Antigen presentation occurs by secretion of exosomes containing membrane Major Histocompatibility Complex (MHC) molecules, which are recognised by T cell receptors and provoke their activation. Also, exosomes released by DCs that have recognised an antigen, carry the antigen to other DCs. Respectively, T helper cells activate B cells leading to increased secretion of exosomes containing MHC complexes to their membrane. In particular, it has been shown that exosomes secreted by B cells activate CD4+ T cells, which undermines the crucial role of exosomes in modulating the immune response. Upon completion of the immune response, exosomes are released by the DCs whose role is to promote its suppression, granted that they stimulate T cell apoptosis and lead to the conversion of T helper cells into regulatory T lymphocytes, thereby balancing pro-inflammatory and anti-inflammatory cells (Corrado et al., 2013).

Another crucial role of exosomes is in the brain and nervous system functions. Here, exosomes participate in and assist the communication of neural cells with other types of cells, mainly between cells responsible for nerve axis integrity and myelination. Additionally, communication between neurons and oligodendrocytes, which are involved in the myelinating process, also depends on the secretion of exosomes. According to this mechanism, secretion of exosomes is signalled by glutamate, which as a neurotransmitter, leads to activation of glial ionotropic glutamate receptors. Through this process, exosomes are internalised by the neurons and release their cargo, which is now available for use. It has been reported that oligodendrocytic exosomes contain enzymes that resist oxidative stress, such as catalases and superoxide dismutase-1, thereby increasing neuronal tolerance to oxidative stress (Fruhbeis et al., 2013; Frohlich et al., 2014). Some studies have also recorded higher activation and expression of signalling pathways, such as the AKT and ERK pathways, in neurons that internalise exosomes (Frohlich et al., 2014).

In the cardiovascular system, exosomes have been observed to contain TNF-α in hypoxic conditions (Yu et al., 2012). Cardiomyocytes secrete exosomes with their secretion increasing rapidly under hypoxic conditions, while their contents change (Gupta and Knowlton, 2007). Under normal circumstances, no production of TNF-α is present in the heart tissue, as opposed to hypoxia. In this case, this factor is produced and secreted by the cardiomyocytes and transferred via exosomes to other healthy cells in which it induces apoptosis (Yu et al., 2012). Thus through this mechanism, exosome secretion by the cells under stress conditions leads to the propagation of an inflammatory reaction. Exosomes also have the potential to induce modifications in the gene expression of recipient cells due to the genetic material, DNA and RNA, that can be transferred through them (Waldenstrom et al., 2012). Therefore, exosomes constitute a non-specific cell type way of communicating in the heart (Danielson and Das, 2014).

The role of exosomes is also important in pathological situations, where they are involved in the development and spread of diseases. Their role has been clarified mainly in neurodegenerative (Vella et al., 2008) and cardiovascular diseases (Halkein et al., 2013), liver disease (Masyuk et al., 2013) and cancer (Hannafon and Ding, 2013).

In neurodegenerative diseases, such as Parkinson and Alzheimer, exosomes may be responsible for disease spread. In Alzheimer disease (AD), accumulation of amyloid β (Aβ) molecules takes place, resulting in plaque formation in the brain (Bellingham et al., 2012). In this particular case, exosomes are involved in the transfer of amyloid β molecules to other neural cells of the brain resulting in the local spread of the disease. Also, increased secretion has been observed through the detection of an exosomal marker, Alix, in the brain of people with AD, as opposed to healthy ones in whom this marker is not detected (Aguzzi and Rajendran, 2009). Similarly, in Parkinson disease, α-synuclein enters exosomes that provide a catalytic environment through their lipids content. The result is a faster transfer of α-synuclein to other neural cells and consequent accumulation in the brain (Grey et al., 2015).

A similar function of exosomes also occurs in cases of heart failure, and a more specific example is peripartum cardiomyopathy (PPCM), which occurs in pregnant or postpartum women. In this particular condition, exosomes function as carriers of a specific miRNA, miRNA-146a, which is produced by a prolactin fragment. These exosomes are taken up by cardiomyocytes and release their content into these cells, causing a decrease in cell metabolic activity and alterations in gene expression, ultimately resulting in heart failure (Halkein et al., 2013).

At the heart of the research on exosomes is the study of their role in cancer. The exosomes secreted by cancer cells are transported to other cells of the same or other tissues, transferring both genetic material and proteins, that cause tumour proliferation and metastasis (Iero et al., 2008; Hood et al., 2009; Hood et al., 2011; Kalluri, 2016; Steinbichler et al., 2017; Whiteside, 2017). Examples are several types of cancers such as prostate and breast cancer in which proteins that induce fibroblast differentiation into myofibroblasts are transported through exosomes (Webber et al., 2010; Vong and Kalluri, 2011) into different cells to activate Wnt signalling and cause activation and increased motility and activation of cancer cells resulting in metastases mainly to the lungs (Luga et al., 2012; Kahlert and Kalluri, 2013). Similar in vitro studies have been conducted to study the promotion of metastasis by components of the exosomes (Jung et al., 2009; Grange et al., 2011). One example is the case in which exosomes from melanoma cells promote bone marrow cell tumorigenesis and metastases (Peinado et al., 2012). Finally, there is a role of exosomes secreted by cancer cells in causing immunosuppression, which leads to suppression of the T cell response (Chalmin et al., 2010). Exosome RNA content

Aside from DNA, proteins and lipids, exosomes possess a substantial RNA content. RNA species, such as messenger RNA (mRNA), miRNA, and long ncRNA (lncRNA) were shown to be present in exosomes in multiple studies. More modern techniques have revealed the presence of additional RNA species within exosomes, such as small nuclear RNA (snRNA), piwi-interacting RNA (piRNA), vault RNA, transfer RNA (tRNA), small nucleolar RNA (snoRNA), Y-RNA, SRP-RNA, small conditional RNA (scRNA), 7SK-RNA, as well as fragmented RNAs (Turchinovich et al., 2019). Moreover, certain modifications of exosomal RNA, such as the 3’-end nucleotide additions and the 5’-terminal oligopyrimidine, have been reported and are possibly tied to RNA quality control processes (Koppers-Lalic et al., 2014; Baglio et al., 2016).

RNAs transcribed in a cell and released into an exosome can be received by another cell, resulting in the transfer of the RNA to the recipient cell in its functional form. This process has been described by various studies over several years, while more recent evidence has shed light on the mechanisms of RNA loading into the exosome. More precisely, RNA-binding proteins appear to bind specific subsets of RNAs. An example of this mechanism is the function of Gag and Gag-like proteins. These proteins impact the RNA content of the exosome by binding genomic RNA and other RNAs and transferring them into exosomes (Ashley et al., 2018; Pastuzyn et al., 2018). This exosomal transfer of RNA can play a crucial role in severe pathological conditions, such as cancer progression and metastasis.

Distribution of lncRNAs in exosomes has been strongly related to the parent cell type (Chen et al., 2016) while also being subjected to regulation by changes in the cellular environment and possibly involved in disease pathogenesis (Hewson et al., 2016). Studies have also shown that lncRNAs contained within exosomes can impact the function of cellular proteins involved in cell signalling, nucleosomal architecture and cell metabolism. Notably, several lncRNAs observed within exosomes have been found to function in cancer cell signalling (Hewson et al., 2016). Kogure *et al.* reported that the exosomal lncRNA TUC339 from liver cancer cells could affect the microenvironment of the tumour, resulting in changes in adhesion and growth of tumour cells through the horizontal information transfer via exosomes (Kogure et al., 2013). Another study shed light on the effect of lncRNA on gastric cancer cells, pointing out that the transfer of lncRNA ZFA1 through exosomes promoted the progression of this type of cancer (Pan et al., 2017).

MiRNAs, the best-known class of RNA exosomal content, have been described by many studies as biomarkers and important components in intercellular communication. It has been shown that the proportion of miRNA is higher within exosomes than within the parent cell (Goldie et al., 2014). As not all miRNAs are present in exosomes and changes in the cellular environment regulate their export, it has been speculated that specific miRNAs exit the cell in a tightly controlled process (Perez-Boza et al., 2018).

MiRNAs within the exosome, function in two broad ways. One is the conventional negative regulation leading to changes in the expression of target genes. The second, more recently described function comes into view when miRNAs function is observed in their exosomal rather than intracellular state. Such studies were conducted on miR-29 and miR-21 contained in cancer cell-secreted exosomes, which were found to possess the ability to act as ligands, activating immune cells via Toll-like Receptor (TLR) binding (Fabbri et al., 2012).

Several subspecies of miRNAs with roles in exocytosis, hematopoiesis tumorigenesis, and angiogenesis have been documented in intercellular communication via exosomes (Waldenstrom and Ronquist, 2014). Oshima *et al.* reported different levels of specific miRNA populations in exosomes derived from different cancer cell lines (Ohshima et al., 2010). Moreover, different levels of specific miRNAs were reported in exosomes from the serum of healthy individual and glioblastoma patients (Skog et al., 2008). Similar differences in specific exosomal miRNA levels have been reported between ovarian cancer and benign tumour cells (Taylor and Gercel-Taylor, 2008) as well as between exosomes from the plasma of healthy individuals and of non-small-cell lung carcinoma patients (Silva et al., 2011).

Lastly, another study reported a close relationship between the expression of miR-134, a microRNA found in exosomes, and breast cancer, suggesting that this miRNA species can be used as a biomarker for diagnosis as well as a possible target for drug therapy (O’Brien et al., 2015).

## Exosome Applications

Interest in exosome research has escalated in the last decade because of their potential therapeutic applications (Li et al., 2019). Exosomes may be indeed used as biomarker resources and as therapeutic biomolecule carriers (Zhang et al., 2019). The key exosome feature that can be exploited is the fact that different cell types display differences in their exosome cargo (Sancho-Albero et al., 2019). For instance, it has been demonstrated that exosomal miRNAs that partake in essential biological functions are lineage-specific and can override specific physiological mechanisms, and thus have the potential for a variety of clinical uses (Narayanan et al., 2018).

Biomarkers are accurate and measurable indicators of health or pathological state (Comabella and Montalban, 2014). Biomarkers may include DNA, RNA, proteins, and metabolites. A particular non-invasive procedure of identifying biomarkers is the use of bodily fluids, such as serum, plasma, saliva and urine. Monitoring proteins in bodily fluids such as plasma, though, is a difficult procedure in complex disorders like cancer owing to the dynamic range of proteins contained, which may obstruct the detection of low abundance proteins. A promising way to overcome such difficulties is the use of EVs found in biological fluids, in particular exosomes (Boukouris and Mathivanan, 2015). As mentioned above, exosome cargo can provide extensive information on the state of the parental cell. Since pathological conditions lead to cells manufacturing disease-associated products, exosomes could contain a specific number of these products.

Furthermore, pathogens like viruses can take advantage of exosomes to infect host cells (Isola and Chen, 2017). Thus, assays for disease-associated molecules contained in exosomes may provide a high specificity biomarker test. The use of exosomes has many advantages compared to traditional biomarker tests because they are less complex samples than the whole bodily fluids, and their cargo is highly stable in storage conditions (Boukouris and Mathivanan, 2015). Current studies have showcased the potential of using exosomes as biomarkers in cancer prognosis and diagnosis, but more research is needed to evaluate the feasibility of such tasks (Huang and Deng, 2019; Jalalian et al., 2019; Wong and Chen, 2019).

Naturally occurring exosomes could also be used as therapeutic biomaterials (Conlan et al., 2017) because they may have therapeutic abilities characteristic of their counterpart live cells. Significant examples are mesenchymal stem cell (MSCs)derived exosomes (Zhao et al., 2019). These cells are used as cellular therapy due to their regenerative and immunomodulatory effects. Granted that the vital mechanism behind mesenchymal stem cells derives from their paracrine ability, it is thought that various factors contained in their respective EVs orchestrate the main actions of MSCs (Hong et al., 2019). The use of such exosomes may reduce side effects, including infusional toxicity (Mendt et al., 2019).

Moreover, naturally occurring exosomes could be used as biomolecule carriers (Akuma et al., 2019). There are various methods to load exosomes with the desired biomolecules and target specific cells. In the case of miRNAs, they can be loaded into exosomes through several methods including transfection of isolated exosomes with commercialised reagents, electroporation, active packaging through the use of proteins or conserved sequences of exosome enriched RNAs (eRNAs), transfection of the parental cells and the production of hybrid exosomes with liposomes (Liu and Su, 2019). In the case of small molecules, like chemotherapy drugs, loading methods may include direct mixing, ultrasonic treatment, and incubation with parental cells (Liu and Su, 2019). Regarding the targeting specificity of these exosomes, it can be determined through the selection of distinct parental cells, construction of targeting molecules or chemical modifications on the exosome surface (Liu and Su, 2019). All these methods have as a final goal the transport of therapeutic molecules to pathological cell targets and can be potentially applied as a therapeutic possibility to a large and diverse number of diseases (Samanta et al., 2018). In the case of cardiovascular diseases, MSC-derived exosomes could be potentially applied. A study in mice showcased that purified MSC-derived exosomes can mitigate complications caused by reperfusion injury in myocardial ischemia after surgical blood flow restoration (Goran Ronquist, 2019). Specifically, the administration of MSC-derived exosomes just before reperfusion restores ATP and NADH levels while simultaneously reduce oxidative stress. Exosomes have also been proposed as therapeutic biomolecules for autoimmune diseases by exploiting their ability as immunomodulatory agents. In type 1 diabetes mellitus, SMCs might protect pancreatic islets of patients from autoimmune targeting and therefore slowing disease progression (Xu et al., 2019). In neurological, immune disorders, exosomes could deliver anti-inflammatory drugs to target brain cells. In a particular study, exosomes used to encapsulate curcumin or an inhibitor of the signal transducer and activator of transcription 3 (stat3) were noninvasively delivered to microglia cells and induced the apoptosis of the targeted microglial cells. This strategy could delay experimental autoimmune encephalomyelitis, an animal model of multiple sclerosis progression in mice (Zhuang et al., 2011). Another autoimmune disease that could provide a potential use for therapeutic exosomes is rheumatoid arthritis. A research study has shown that IL-10-treated dendritic cells-derived exosomes may be able to suppress the onset of murine collagen-induced arthritis, an animal model of rheumatoid arthritis, as well as to reduce the severity of established arthritis (Kim et al., 2005). Exosomes could also help diagnose or even be a potential treatment for developmental brain disorders. Specifically, in Rett syndrome, a developmental brain disorder with autismlike symptoms, Rett-affected exosomes lack essential neurodevelopmental proteins, while the administration of ‘healthy’ exosomes to a culture-dish model of Rett syndrome displayed therapeutic effects (Sharma et al., 2019). Lastly, exosomes have been thoroughly studied for their therapeutic application in cancer. It has been shown that dendritic cell-derived exosomes can prime naïve T-cells and activate natural killer cells to shrink tumours (Gao and Jiang, 2018). Moreover, exosomes can deliver synthetic anticancer drugs to targeted cancer cells (Lu et al., 2018).

While the clinical use of naturally occurring exosomes seems to be an up-and-coming field of study, it is important to be prescient in their utilisation as they take part in a large number of physiological pathways. Their multifaceted abilities might have adverse effects on a patient’s immune response, cancer progression, drug resistance and metabolism (Conlan et al., 2017). Furthermore, several challenges may also arise due to the difficulty in production, isolation, and storage on a commercial scale (Yamashita et al., 2018). These difficulties can be addressed through the production of synthetic exosome-like biomaterials (Garcia-Manrique et al., 2018). However, although natural exosomes clinical trials have just begun, synthetic exosomes are still in their first steps, demanding the development of standardised production protocols, studying their modes of actions and performing safety checks (Garcia-Manrique et al., 2018).

## Concluding Remarks

Exosomes are an intriguing field of study. Their cargo and unique abilities imply vast potential in their use as biomarkers, natural therapeutic vehicles and drug carriers. Before advancing in their clinical application, though, the mechanisms dictating their role in physiological and pathological conditions should be better elucidated.

## Key Points

- Exosomes are single-membrane nano-sized vesicles secreted by a great variety of cell types that have an essential role in intercellular signaling both in physiological and pathological conditions.
- Exosomes are carriers of various biomolecules such as DNA, RNA, lipids, and proteins, with mentioned cargo characterizing their function.
- miRNAs, a class of non-coding RNAs, are the best-known class of RNA exosomal content and may influence gene expression and immune response.
- Exosomes can be used as biomarkers, where their cargo can provide extensive information on the state of the parental cell.
- Exosomes can also be used as therapeutic biomaterials, where their natural cargo or artificially loaded biomolecules can provide them with therapeutic abilities.
